# Self-quarantining, social distancing, and mental health during the COVID-19 pandemic: A multi wave, longitudinal investigation

**DOI:** 10.1371/journal.pone.0298461

**Published:** 2024-02-26

**Authors:** Jerin Lee, Jenna Wilson, Benjamin Oosterhoff, Natalie J. Shook

**Affiliations:** 1 University of Connecticut, Storrs, CT, United States of America; 2 Brigham and Women’s Hospital, Boston, MA, United States of America; 3 Meadows Mental Health Policy Institute, Dallas, TX, United States of America; University of Connecticut Health Center: UConn Health, UNITED STATES

## Abstract

Social isolation and disconnectedness increase the risk of worse mental health, which might suggest that preventive health measures (i.e., self-quarantining, social distancing) negatively affect mental health. This longitudinal study examined relations of self-quarantining and social distancing with mental health during the COVID-19 pandemic. A U.S. national sample (*N* = 1,011) completed eight weekly online surveys from March 20, 2020 to May 17, 2020. Surveys assessed self-quarantining, social distancing, anxiety, and depression. Fixed-effect autoregressive cross-lagged models provided a good fit to the data, allowing for disaggregation of between-person and within-person effects. Significant between-person effects suggested those who engaged in more self-quarantining and social distancing had higher anxiety and depression compared to those who engaged in less social distancing and quarantining. Significant within-person effects indicated those who engaged in greater social distancing for a given week experienced higher anxiety and depression that week. However, there was no support for self-quarantining or social distancing as prospective predictors of mental health, or vice versa. Findings suggest a relationship between mental health and both self-quarantining and social distancing, but further longitudinal research is required to understand the prospective nature of this relationship and identify third variables that may explain these associations.

## Introduction

The Coronavirus disease 2019 (COVID-19) pandemic has negatively impacted both physical and mental health. Although effective containment of highly communicable diseases, such as COVID-19, largely depends on limiting social contact [1], there are concerns that such preventive health measures (i.e., self-quarantining, social distancing) may negatively affect mental health, increasing anxiety and depression [2–4]. Though these measures are effective at reducing transmission and illness from COVID-19 [5], self-quarantining and social distancing may prompt feelings of disconnectedness and social isolation [2]. Furthermore, physically distancing from one another may increase opportunities for anxious rumination [6]. Social connectedness benefits mental health [7], whereas social isolation negatively affects health [8]. However, little empirical research has longitudinally examined the proposed relations of self-quarantining and social distancing with mental health. Thus, the goal of the present study was to utilize a multi-wave, longitudinal study design to determine the directional associations of self-quarantining and social distancing with symptoms of anxiety and depression during the COVID-19 pandemic.

### Self-quarantining

Humans are social animals [9], and decades of research link social isolation with worse mental health outcomes [10]. Thus, it may not be surprising that public health measures encouraging social isolation may threaten mental well-being. Self-quarantining policies encourage individuals who have contracted COVID-19 or who may have been exposed to the virus to self-isolate and stay away from other people for several days to monitor symptoms and/or recover [11]. Indeed, some research lends support for an association between self-quarantining and poorer mental health. A review of studies examining the effects of isolation on mental health among individuals quarantined due to infectious diseases (e.g., Middle East respiratory syndrome, Ebola) found positive associations between quarantining and depression, anxiety, irritability, and post-traumatic stress symptoms [12].

In the context of the COVID-19 pandemic, mass-level quarantining (e.g., quarantining enforced at the national level) has been associated with symptoms of anxiety and depression. A meta-analysis of cross-sectional, longitudinal, and experimental studies demonstrated a small positive effect for the association between mass-level quarantine duration and symptoms of anxiety and depression [13]. Further, a meta-analysis of cross-sectional and longitudinal studies examining the impact of national lockdowns (i.e., requirement of citizens to stay home) concluded that the negative effect of lockdown measures on anxiety and depression in the general population is small in magnitude [14]. The results of both meta-analyses challenge claims that COVID-19 lockdowns may have had a considerable negative effect on population mental health. However, both meta-analyses focused on mass quarantine measures (i.e., mass-level quarantine duration, assessment of mental health before and after lockdown orders, comparing regions with and without lockdown orders), which limits conclusions regarding the individual-level self-quarantining (i.e., isolating oneself from others to recover from and/or avoid spreading COVID-19).

Regarding self-quarantining at the individual level, findings are mixed and few. Cross-sectional research conducted in various countries (e.g., Albania, Argentina, China, Hong Kong) demonstrated that self-quarantining was positively associated with anxiety and/or depressive symptoms [15–17]. However, other cross-sectional studies conducted in the U.S. suggest self-quarantining is not significantly associated with anxiety and depressive symptoms [18, 19]. Further, in a recent two-wave longitudinal study that assessed three independent U.S. samples, self-quarantining was not associated with changes in anxiety and depressive symptoms from pre-pandemic to during the pandemic [20]. To date, literature regarding associations between self-quarantining during COVID-19 and mental health is limited to mixed findings from cross-sectional and two-wave longitudinal studies.

### Social distancing

Social distancing involves maintaining a distance of 6 feet from others and avoiding crowded and poorly ventilated establishments [21]. Social distancing may disrupt social processes that are often beneficial to mental health, such as day-to-day interactions and the availability of social support [22]. Although quarantining has been studied in the context of past pandemics, social distancing policies are unique to the COVID-19 pandemic, and existing evidence regarding the link between social distancing and mental health is mixed. Cross-sectional studies demonstrated that greater social distancing was associated with worse anxiety and/or depressive symptoms in samples recruited from Germany [23] and the U.S. [22, 24, 25]. Yet, other cross-sectional research indicates that greater social distancing was associated with less anxiety and depressive symptoms in a sample from Hong Kong [17]. Moreover, some cross-sectional [18, 19] and two-wave longitudinal [20] studies demonstrated that social distancing is not significantly associated with anxiety and depressive symptoms in U.S. adults. Such findings are inconsistent with predictions that physically distancing from one another would be associated with poorer mental health [4, 26].

### Methodological limitations of existing research

There are several methodological limitations associated with existing studies examining the association between self-quarantining, social distancing, and mental health. First, the extant literature is primarily limited to cross-sectional (single time point) study designs. Cross-sectional research can provide between-person data that speak to associations across a *set* of individuals; however, conclusions about fluctuations in variables *within* an individual (e.g., are changes in self-quarantining associated with changes in anxiety within a person) cannot be made [27].

Second, two-wave longitudinal designs are prone to confounding variables and little can be concluded about *how* change occurs [28] or the directionality of associations. From two time points, it cannot be discerned which variable changes first or whether there is a bidirectional association. Indeed, it is possible that self-quarantining or social distancing may have a bidirectional relationship with mental health, such that those with higher levels of anxiety and/or depression are more likely to engage in social distancing and self-quarantining. Theoretically, avoidance is a key component of anxiety [29]. Thus, highly anxious individuals who are concerned about contracting COVID-19 may be more likely to engage in preventive behaviors. Consistent with this notion, Velikonja and colleagues [30] found that anxiety was associated with a broad measure of preventive measures that included social distancing. Furthermore, theories of depression suggest that behavioral withdrawal is a core component of depression that may serve to regulate mood [31] or provide short-term relief from potentially aversive experiences [32]. Thus, people experiencing high levels of depression may be more prone to engaging in behavioral withdrawal, such as self-quarantining or social distancing. No study to date has examined the potentially reciprocal prospective effects between self-quarantining, social distancing, and mental health.

Third, existing research largely focused on mass-level quarantining rather than individual-level engagement, which limits understanding of how self-quarantining and mental health vary within an individual. To address these limitations, repeated measurement from individuals over at least three time points is recommended [33]. Thus, given the inconsistencies in the literature and the infrequent nature of pandemics, more multi-wave longitudinal studies are necessary to provide a better understanding of how self-quarantining and social distancing may be associated with mental health to better inform interventions for future pandemics.

### Present study

On March 11, 2020, the World Health Organization declared COVID-19 a pandemic [34], prompting many countries, including the United States, to implement self-quarantining and social distancing policies to reduce the spread of the disease. On March 15–16, 2020, states across the U.S. shut down schools and other public areas (e.g., restaurants, bars) and the White House issued social distancing guidelines to prevent the spread of COVID-19 [34, 35]. On April 24, 2020, some U.S. states began to partially reopen [34]. The goal of the present study was to assess fluctuations in variables *between* and *within* individuals, and longitudinally assess the extent to which self-quarantining and social distancing prospectively predicted mental health (i.e., anxiety and depression), and vice versa, over eight weeks in a national sample of U.S. adults from March 20, 2020 to May 17, 2020. This timeframe is uniquely marked by the introduction of self-quarantining and social distancing policies in March 2020 and the loosening of these policies by May 2020. Thus, we would expect this novel and transitional period (as opposed to a period where policies have been in place for longer) to be associated with changes in self-quarantining, social distancing, and psychological adjustment.

We expected greater days of quarantining per week to be associated with greater anxiety and depressive symptoms. Although there are mixed findings regarding social distancing, we hypothesized that greater social distancing per week would be associated with worse mental health due to the shared feature of social disconnection present in both self-quarantining and social distancing. Furthermore, given avoidance and behavioral-based theories of anxiety and depression, respectively, we hypothesized that greater anxiety and depression would predict greater engagement in self-quarantining and social distancing.

## Materials and methods

### Participants and procedure

Participants were recruited through the panel provider Qualtrics for a larger 29-wave longitudinal study about the effects of COVID-19. For the purposes of the present study, only the first eight waves were examined. A minimum sample of 500 was estimated using Monte Carlo simulations (N  =  10,000) for the larger study to provide sufficient power (>95%) to detect anticipated effects (β = .15 to .20) assuming α = .05. To account for missing or unusable data, at least 1,000 U.S. individuals were recruited. Data were collected every week for eight weeks from a national sample of 1,213 U.S. residents from March 20, 2020 to May 17, 2020. Two hundred two participants were excluded due to atypical response patterns (e.g., open-ended responses unrelated to the prompt, straightline responses) or failure to complete at least 60% of either the first survey or primary study variables. It was not possible to meaningfully examine group differences between participants that were included and excluded in the present analyses because all of the 202 excluded participants discontinued the survey before providing demographic variables. Additionally, primary study variable data were only available for 1 to 51 participants. The final sample comprised 1,011 participants (51% women, *M*_age_ = 46.6 years, *SD*_age_ = 16.5, range: 18–85 years; 76.1% White, 7.1% Asian, 6.2% Black, 3.7% Latinx, 3.4% more than one race, 0.6% “Other,” 0.4% Native American; *Mdn*_education_ = College graduate; *Mdn*_income_ = $70,000-$79,999).

Institutional Review Board (IRB) approval was obtained from the first author’s institution (Protocol #L20-0018). As the study was conducted online and anonymously, written consent was not feasible and consent was requested electronically. The IRB approved use of electronic consent. Participants read and acknowledged an online consent form and indicated their consent by clicking on an "Agree" radio button. Those who clicked "Disagree" were automatically exited out of the Qualtrics survey. Participants completed primary study measures and other questionnaires in a random order, with one exception. Demographics were assessed at the end of the Wave 1 survey. Self-quarantining was not assessed at Wave 1 but was included in the Wave 2 through 8 surveys. Surveys were sent out on a weekly basis, and each online survey took approximately 25 minutes. Participants were provided monetary compensation in an amount established by the panel provider. Preregistration information and research materials (e.g., data, code) are publicly available (https://osf.io/56fcv?view_only=002af22544b547c387624f726486311b and https://osf.io/s9uxv/?view_only=290032cc1ec94381981fda8858ee1180).

### Measures

A list of measures that were included as part of the larger study is in S1 Appendix. The present study focused on the measures below.

#### Self-quarantining

Starting with the second wave, participants indicated (“Yes” or “No”) whether they self-quarantined (i.e., stay at home to avoid contracting or spreading COVID-19) over the past week [20]. For individuals who responded “Yes,” they were asked to indicate how many days in the past week they had self-quarantined (1 to 7 days). A continuous variable was created by coding “No” responses as 0 and utilizing the number of days self-quarantined in the past week for “Yes” responses.

#### Social distancing

Six items assessed social distancing [36]. Participants indicated the extent to which they engaged in five items related to avoiding contact with others (e.g., ‘Avoid going to school/job,’ ‘Avoid hugging someone for greeting’) within the past week on a scale ranging from 1 (*Not at all*) to 6 (*Multiple times a day*). Additionally, participants reported the extent to which they engaged in social distancing in the past week on a scale ranging from 1 (*Not at all*) to 5 (*A great deal*). This measure of social distancing has demonstrated acceptable reliability in prior research (α = .76) [36]. All items were standardized and averaged to create a composite score. Higher scores indicate more social distancing. This measure demonstrated acceptable reliability across all eight waves within our sample (αs = .75 –.78).

#### Anxiety symptoms

The Generalized Anxiety Disorder screener (GAD-7) [37] is a 7-item measure of anxiety. Participants indicated their experience of anxiety symptoms (e.g., ‘Feeling nervous, anxious, or on edge’) over the past two weeks for the first wave and over the past week for subsequent waves using a scale ranging from 0 (*Not at all*) to 3 (*Nearly every day*). Scores are summed and range from 0–21, with the following severity ratings: minimal to no anxiety symptoms (0–4), mild (5–9), moderate (10–14), and severe (15+). Scores of ≥10 suggest presence of GAD. The GAD-7 has demonstrated good reliability in clinical (α = .92) [37] and community-based samples (α = .89) [38]. Prorated scores were computed to account for individuals who missed two or fewer items [39]. The GAD-7 demonstrated strong reliability across all eight waves within our sample (αs = .95 –.96).

#### Depressive symptoms

The abbreviated 8-item version of the Patient Health Questionnaire (PHQ-9) [40] assessed depressive symptoms. One item about suicidal ideation was excluded from this study. Participants indicated their experience of depressive symptoms (e.g., ‘Feeling down, depressed, or hopeless’) over the past two weeks for the first wave and over the past week for subsequent waves using a scale ranging from 0 (*Not at all*) to 3 (*Nearly every day*). Scores are summed and range from 0–24, with the following severity ratings: minimal (0–4), mild (5–9), moderate (10–14), moderately severe (15–19), and severe (20–24). Scores of ≥10 suggest presence of major depression. The PHQ-9 has demonstrated strong internal reliability (α = .89) in prior research [40]. Prorated scores were computed to account for individuals who missed two or fewer items [41]. The abbreviated PHQ-9 demonstrated strong reliability across all eight waves within our sample (αs = .93 –.94).

### Data analytic plan

Fixed-effect autoregressive cross-lagged (FACL) structural equation models were used to examine the extent to which self-quarantining, social distancing, anxiety, and depression covary within individuals over time. FACL models can isolate within-person effects from between-person effects with cross-lagged data [27]. Between-person effects provide information regarding how variables may differ between individuals (e.g., Do people who engage in more self-quarantining report greater depressive symptoms relative to those who engaged in less self-quarantining?). Within-person effects provide information regarding how variables change *within* an individual over time (e.g., Is engaging in more self-quarantining one week compared to one’s average level of self-quarantining associated with greater anxiety during the same week compared to one’s average level of anxiety?). Lastly, cross-lagged effects provide information regarding the directional effect of two variables across time (e.g., Does self-quarantining prospectively predict greater depressive symptoms or vice versa?).

Latent intercepts were fixed for each construct, which specified mean scores for week-level self-quarantining, social distancing, and mental health. Autoregressive and cross-lagged paths were specified among the residuals of the weekly indicators. Consistent with prior research [27], the variances of the residuals were constrained to be equal. For parsimony, we also constrained each autoregressive and cross-lagged effect to be equal given that we did not expect differences in these estimates across weeks. Covariances were specified across constructs for the same week and were constrained to be equal to indicate weekly effects. A covariance was also specified across intercepts for the latent variables and was used to indicate an overall between-person effect.

Attrition varied across waves, as individuals could complete surveys even if they missed a particular week. Attrition (*n*) at each wave was as follows: Wave 1 (0), Wave 2 (190), Wave 3 (237), Wave 4 (262), Wave 5 (287), Wave 6 (317), Wave 7 (360), and Wave 8 (351). Little’s test of Missing Completely at Random (MCAR) for each of the eight waves was not significant, *p*s = .16 - .78, indicating that there was no evidence supporting the hypothesis that the data were not MCAR. Thus, missing data were assumed to be missing at random and estimated using multiple imputation [42]. Analyses were conducted with imputed and non-imputed data. All non-imputed data results are in S2, S3 and S5–S7 Tables.

Analyses were conducted using the lavaan package [43] in RStudio. Given that chi-square tests often inflate Type I error rates in large samples [44], acceptable model fit was evaluated with the Comparative Fit Index (CFI) ≥ .95, Tucker-Lewis Index (TLI) ≥ .95, Root Mean Square Error of Approximation (RMSEA) ≤ .06, and Standardized Root Mean Square Residual (SRMR) < .08 [45].

## Results

Means and standard deviations are presented in Table 1. Within this sample, clinical cutoffs for generalized anxiety and major depression were calculated. At Wave 1, 27.2% of participants reported clinically significant anxiety, followed by 23.6%, 23.3%, 24.1%, 20.6%, 19.1%, 16.9%, and 18.5% for subsequent waves. Additionally, 25.6% of participants reported clinically significant depression at Wave 1, followed by 25.0%, 25.7%, 21.4%, 22.0%, 22.5%, 19.8%, and 19.7% for subsequent waves. Data regarding anxiety and depression severity are provided in S1 and S2 Tables.

**Table 1:** Means (standard deviations) for repeated-measures ANOVAs.

	Wave 1	Wave 2	Wave 3	Wave 4	Wave 5	Wave 6	Wave 7	Wave 8
Self-quarantining^a^	---	4.70 (2.86)^7,8^	4.72 (2.86)^6,7,8^	4.79 (2.87)^5,6,7,8^	4.61 (2.90)^4,7,8^	4.51 (2.97)^3,4,7,8^	4.35 (3.00)^2,3,4,5,6,8^	4.14 (2.98)^2,3,4,5,6,7^
Social Distancing	-0.01 (0.68)^6^	0.00 (0.67)^6^	0.02 (0.69)^7^	0.02 (0.67)^7^	0.01 (0.70)^7^	0.03 (0.70)^1,2,7^	-0.03 (0.69)^3,4,5,6,8^	0.02 (0.70)^7^
Anxiety	6.13 (5.98)^2,3,4,5,6,7,8^	5.58 (5.89)^1,5,6,7,8^	5.62 (5.92)^1,4,5,6,7,8^	5.38 (5.86)^1,3,5,6,7,8^	4.96 (5.76)^1,2,3,4,6,7,8^	4.64 (5.60)^1,2,3,4,5,7^	4.40 (5.43)^1,2,3,4,5,6^	4.55 (5.66)^1,2,3,4,5^
Depression	5.73 (6.11)^4,5,6,7,8^	5.53 (6.06)^3,5,6,7,8^	5.78 (6.37)^2,4,5,6,7,8^	5.28 (6.07)^1,3,6,7,8^	5.11 (6.08)^1,2,3,7,8^	5.00 (6.03)^1,2,3,4,7^	4.73 (5.92)^1,2,3,4,5,6^	4.79 (5.99)^1,2,3,4,5^

*Note*. Superscripts indicate significant mean differences between weeks (e.g., 7,8 above Wave 2 for self-quarantining indicates that the mean for Wave 2 self-quarantining is significantly higher than the means for Waves 7 and 8 self-quarantining).

^a^ Self-quarantining was not assessed at Wave 1.

^b^ The social distancing composite reflects six items that were standardized and averaged to have a mean of 0 and standard deviation of 1.

### Changes over time

**Fig 1:**
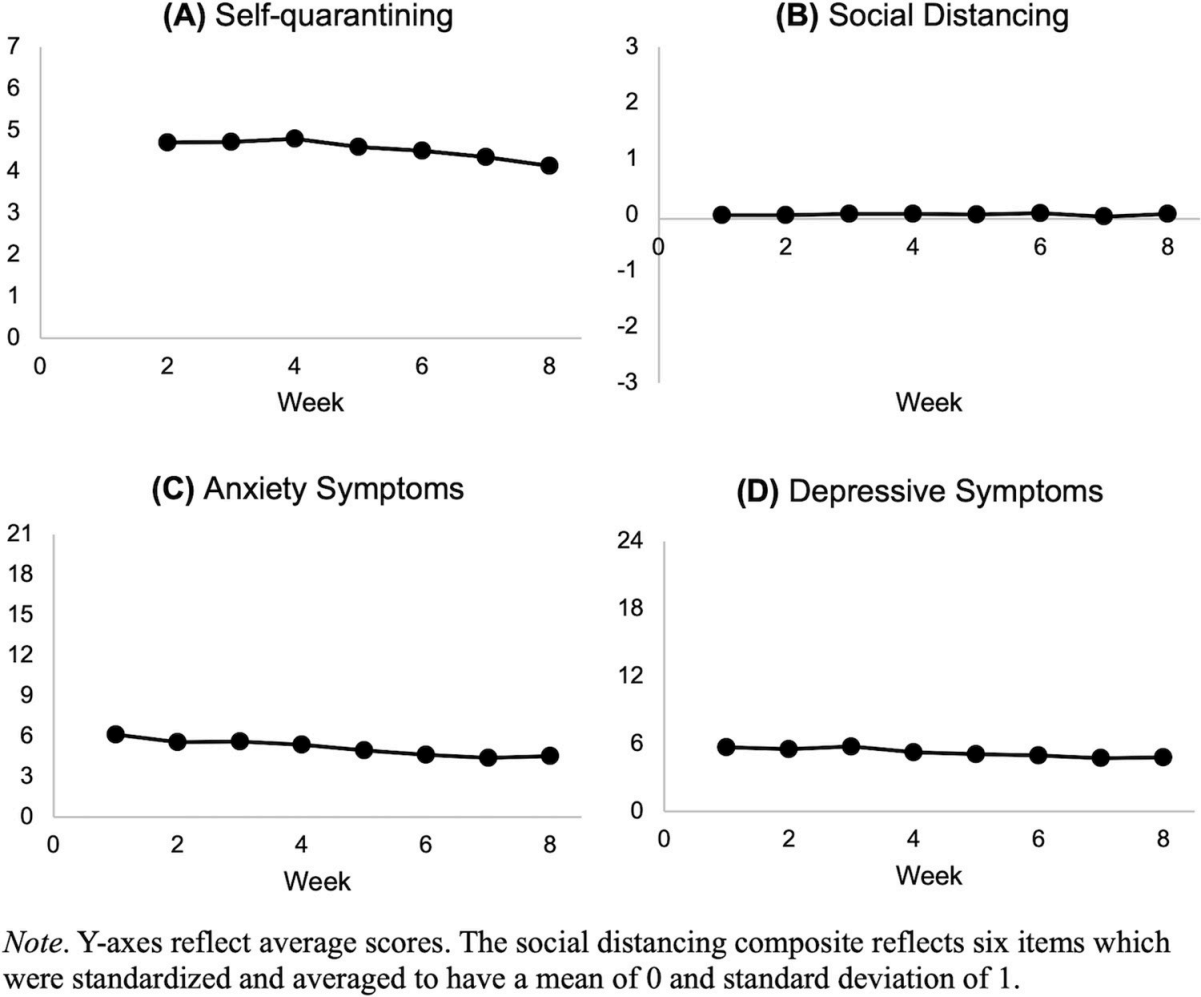
Visual representation of repeated measures ANOVA assessing changes in self-quarantining, social distancing, anxiety symptoms, and depressive symptoms. *Note*. Y-axes reflect average scores. The social distancing composite reflects six items which were standardized and averaged to have a mean of 0 and standard deviation of 1.

To examine changes in self-quarantining, social distancing, anxiety, and depressive symptoms over time, four repeated-measure ANOVAs were conducted (Fig 1 and Table 1). For each ANOVA, time (seven or eight weekly time points) was the within-subjects variable. Repeated measures ANOVA indicated a main effect of time for self-quarantining (*F*(5.43, 5483.19) = 14.43, *p* < 0.001, η_p_^2^ = 0.014), social distancing (*F*(6.45, 6509.84) = 2.84, *p* = 0.008, η_p_^2^ = 0.003), anxiety symptoms (*F*(5.85, 5907.07) = 47.28, *p* < 0.001, η_p_^2^ = 0.045), and depressive symptoms (*F*(6.19, 6249.17) = 19.77, *p* < 0.001, η_p_^2^ = 0.019). Self-quarantining, anxiety symptoms, and depressive symptoms generally decreased over time. Levels of social distancing remained relatively consistent across the eight waves, except for Wave 7, where social distancing was relatively lower than other waves. Patterns of results were similar for the non-imputed data, except for social distancing such that the main effect of time for social distancing was not significant, *F*(6.36, 2703.26) = 1.13, *p* = 0.34, η_p_^2^ = 0.003.

### Correlations

Bivariate correlations assessed associations among key variables across all waves (Tables 2 and 3). Correlations for all variables are in S4 and S5 Tables. Greater self-quarantining was generally significantly associated with greater anxiety and depressive symptoms within and across all waves. Greater social distancing was significantly associated with greater anxiety symptoms and greater depressive symptoms within and across all waves. For non-imputed data, patterns were comparable to patterns resulting from imputed data except self-quarantining was not consistently significantly associated with anxiety and depressive symptoms.

**Table 2:** Bivariate correlations between preventive measures and symptoms of anxiety.

	Anxiety W1	Anxiety W2	Anxiety W3	Anxiety W4	Anxiety W5	Anxiety W6	Anxiety W7	Anxiety W8
Self-quarantining W2	.105***	.078*	.087**	.070*	.065*	.039	.042	.109***
Self-quarantining W3	.150***	.122***	.125***	.086**	.095**	.134***	.118***	.176***
Self-quarantining W4	.137***	.150***	.128***	.088**	.099**	.133***	.113***	.142***
Self-quarantining W5	.109***	.116***	.133***	.100**	.099**	.134***	.105***	.145***
Self-quarantining W6	.123***	.120***	.128***	.095**	.091**	.102***	.071*	.118***
Self-quarantining W7	.046	.096**	.083**	.090**	.065*	.108***	.082**	.092**
Self-quarantining W8	.087**	.090**	.099**	.072*	.074*	.089**	.064*	.133***
Social Distancing W1	.295***	.267***	.248***	.243***	.245***	.268***	.265***	.274***
Social Distancing W2	.202***	.205***	.199***	.175***	.156***	.172***	.188***	.226***
Social Distancing W3	.237***	.208***	.255***	.186***	.214***	.198***	.219***	.230***
Social Distancing W4	.177***	.187***	.207***	.169***	.168***	.165***	.195***	.213***
Social Distancing W5	.197***	.207***	.197***	.190***	.210***	.233***	.233***	.255***
Social Distancing W6	.230***	.207***	.243***	.221***	.199***	.230***	.206***	.249***
Social Distancing W7	.157***	.145***	.204***	.151***	.168***	.174***	.177***	.182***
Social Distancing W8	.204***	.220***	.243***	.217***	.196***	.223***	.215***	.260***

*Note*. *N* = 1,011.

*p < .05.

**p < .01.

***p < .001.

**Table 3:** Bivariate correlations between preventive measures and symptoms of depression.

	Depression W1	Depression W2	Depression W3	Depression W4	Depression W5	Depression W6	Depression W7	Depression W8
Self-quarantining W2	.053	.063*	.061	.071*	.051	.046	.069*	.076*
Self-quarantining W3	.111***	.122***	.116***	.112***	.107***	.096**	.095**	.127***
Self-quarantining W4	.090**	.108***	.101***	.103***	.095**	.091**	.072*	.088**
Self-quarantining W5	.089**	.108***	.112***	.139***	.092**	.112***	.094**	.119***
Self-quarantining W6	.070*	.092**	.097**	.108***	.121***	.054	.037	.099**
Self-quarantining W7	.065*	.070*	.092**	.104***	.070*	.095**	.057	.085**
Self-quarantining W8	.067*	.080*	.078*	.049	.091**	.076*	.044	.092**
Social Distancing W1	.260***	.266***	.247***	.234***	.215***	.246***	.252***	.237***
Social Distancing W2	.196***	.263***	.216***	.182***	.171***	.158***	.187***	.198***
Social Distancing W3	.236***	.230***	.242***	.180***	.203***	.222***	.182***	.194***
Social Distancing W4	.196***	.196***	.221***	.151***	.170***	.166***	.137***	.147***
Social Distancing W5	.219***	.224***	.219***	.176***	.229***	.225***	.198***	.220***
Social Distancing W6	.247***	.229***	.235***	.205***	.218***	.228***	.188***	.218***
Social Distancing W7	.160***	.186***	.195***	.149***	.157***	.166***	.163***	.160***
Social Distancing W8	.241***	.255***	.250***	.232***	.214***	.229***	.223***	.218***

*Note*. *N* = 1,011.

*p < .05.

**p < .01.

***p < .001.

### Self-quarantining and anxiety symptoms

The model testing links between self-quarantining and anxiety symptoms provided a good fit to the data (Table 4). Table 5 provides model estimates. There were significant positive between-person associations, such that those who engaged in greater self-quarantining reported higher anxiety symptoms compared to those who engaged in less self-quarantining. There were no significant weekly within-person effects among self-quarantining and anxiety symptoms, indicating that those who engaged in greater self-quarantining for a given week relative to their own average across the study *did not* report greater anxiety symptoms that week relative to their own average across the study. There was also no evidence of significant cross-lagged effects for engagement in self-quarantining predicting anxiety symptoms the subsequent week, nor anxiety symptoms predicting engagement in self-quarantining the subsequent week. For non-imputed data, between-person, within-person, or cross-lagged effects were not significant for associations between self-quarantining and anxiety symptom.

**Table 4:** Fixed-effect autoregressive cross-lagged model fit statistics.

Model	χ^2^ (df)	CFI	TLI	RMSEA [90% CI]	SRMR
Self-quarantining					
Anxiety symptoms	390.80 (92)	0.97	0.97	0.06 [0.05, 0.06]	0.05
Depressive symptoms	399.43 (92)	0.97	0.97	0.06 [0.05, 0.06]	0.04
Social distancing					
Anxiety symptoms	619.76 (123)	0.97	0.97	0.06 [0.06, 0.07]	0.05
Depressive symptoms	598.49 (123)	0.97	0.97	0.06 [0.06, 0.07]	0.05

*Note*. *N* = 1,011. All *χ*^2^ values are significant at *p* < .001.

**Table 5:** Standardized and unstandardized estimates for models.

	Mental Health							
	Anxiety Symptoms				Depressive Symptoms			
	β	*B*	90% CI	*SE*	β	*B*	90% CI	*SE*
Self-quarantining (SQ)								
SQ ↔ MH Intercept Covariance (Between-person)	0.166	1.764*	[1.019, 2.509]	0.380	0.137	1.554*	[0.764, 2.344]	0.403
SQ ↔ MH Weekly Covariance (Within-person)	-0.004	-0.023	[-0.168, 0.123]	0.074	-0.025	-0.134	[-0.289, 0.022]	0.079
SQ Autoregressive Path	0.19	0.19*	[0.159, 0.222]	0.02	0.19	0.19*	[0.160, 0.223]	0.01
MH Autoregressive Path	0.21	0.21*	[0.176, 0.238]	0.02	0.19	0.19*	[0.156, 0.221]	0.02
SQ → MH Weekly Cross-Lagged	-0.029	-0.040	[-0.079, -0.001]	0.020	-0.017	-0.025	[-0.066, 0.017]	0.021
MH → SQ Weekly Cross-Lagged	-0.005	-0.004	[-0.024, 0.017]	0.010	0.047	0.032*	[0.013, 0.052]	0.010
Social Distancing (SD)								
SD ↔ MH Intercept Covariance (Between-person)	0.300	0.829*	[0.639, 1.019]	0.097	0.287	0.849*	[0.647, 1.051]	0.103
SD ↔ MH Weekly Covariance (Within-person)	0.045	0.048*	[0.021, 0.076]	0.014	0.054	0.060*	[0.031, 0.088]	0.015
SD Autoregressive Path	0.11	0.11*	[0.078, 0.133]	0.01	0.10	0.10*	[0.077, 0.132]	0.01
MH Autoregressive Path	0.23	0.23*	[0.199, 0.254]	0.01	0.18	0.18*	[0.152, 0.207]	0.01
SD → MH Weekly Cross-Lagged	-0.020	-0.142	[-0.321, 0.037]	0.091	-0.010	-0.072	[-0.257, 0.112]	0.094
MH → SD Weekly Cross-Lagged	-0.009	-0.001	[-0.005, 0.002]	0.002	0.003	0.000	[-0.003, 0.004]	0.002

*Note*. *N* = 1,011. MH = Mental Health.

*p ≤ .001

### Self-quarantining and depressive symptoms

The model testing links between self-quarantining and depressive symptoms provided a good fit to the data (Table 4). Table 5 provides model estimates. There were significant positive between-person associations, such that those who engaged in greater self-quarantining reported higher depressive symptoms compared to those who engaged in less self-quarantining. There were no significant weekly within-person effects among self-quarantining and depressive symptoms, suggesting that those who engaged in greater self-quarantining for a given week relative to their own average across the study *did not* report greater depressive symptoms that week relative to their own average across the study. There was also no evidence of significant cross-lagged effects for self-quarantining predicting next week depressive symptoms. However, there was a significant cross-lagged effect for depressive symptoms predicting next week self-quarantining. Those who had higher depressive symptoms one week relative to their eight-week average were more likely to engage in self-quarantining the next week relative to their eight-week average. For example, if Person A had depressive symptoms in Week 3 that was higher than their average level of depressive symptoms across the eight-week study, then they were likely to engage in more self-quarantining in Week 4 than they usually would. For non-imputed data, between-person, within-person, or cross-lagged effects were not significant for associations between self-quarantining and depressive symptoms.

### Social distancing and anxiety symptoms

The model testing links between social distancing and anxiety symptoms provided a good fit to the data (Table 4). Table 5 provides model estimates. There was a significant positive between-person effect, such that those who engaged in greater social distancing reported greater anxiety symptoms compared to those who engaged in less social distancing. There was a significant positive weekly within-person effect between social distancing and anxiety symptoms, indicating that those who engaged in greater social distancing for a given week relative to their own average across the study also experienced greater anxiety that week relative to their own average across the study. For example, if Person B social distanced more in Week 3 compared to their average amount of social distancing across the eight-week study, then they also experienced more anxiety symptoms in Week 3 compared to their average level of anxiety across the eight-week study. There was no evidence of significant cross-lagged effects for social distancing predicting anxiety symptoms the subsequent week or anxiety symptoms predicting social distancing the subsequent week. Patterns of results did not differ for non-imputed data.

### Social distancing and depressive symptoms

The model testing links between social distancing and depressive symptoms provided a good fit to the data (Table 4). Table 5 provides model estimates. There were significant positive between-person associations, suggesting that those who engaged in greater social distancing reported greater depressive symptoms compared to those who engaged in less social distancing. There was a significant weekly within-person effect between social distancing and depressive symptoms, indicating that those who engaged in greater social distancing for a given week relative to their own average across the study also experienced greater depressive symptoms that same week relative to their own average across the study. There were no significant cross-lagged effects for engagement in social distancing predicting depressive symptoms the subsequent week, nor depressive symptoms predicting engagement in social distancing the subsequent week. For non-imputed data, there was a significant positive between-person effect for social distancing and depressive symptoms, and within-person or cross-lagged effects were not significant.

## Discussion

The current study assessed fluctuations in self-quarantining, social distancing, and mental health symptoms (i.e., anxiety and depression) *between* and *within* individuals, as well as prospectively, over eight weeks in a national sample of U.S. adults from March 20, 2020 to May 17, 2020. We found between person effects indicating that those who engaged in greater self-quarantining or social distancing reported higher anxiety and depressive symptoms relative to those who engaged in less self-quarantining or social distancing. Further, we found weekly within-person effects for associations between social distancing, but not self-quarantining, and mental health symptoms. That is, people who engaged in greater social distancing for a given week relative to their own average across the study reported greater symptoms of anxiety and depression that week relative to their own average across the study. Finally, contrary to expectations, we generally did not find evidence for prospective associations between self-quarantining or social distancing with mental health symptoms.

Consistent with concerns and empirical evidence suggesting increases in psychological maladjustment pre- to during the pandemic [20, 46], the present sample reported striking levels of anxiety and depressive symptoms at Wave 1. Within this sample, 27.2% reported clinically significant anxiety symptoms and 25.6% reported clinically significant depressive symptoms. Of note, in a national U.S. adult sample surveyed in 2019, 6.1% reported clinically significant anxiety symptoms [47], and 7.0% reported clinically significant depressive symptoms [48]. These findings speak to the high levels of psychological distress experienced by U.S. adults during the initial weeks of the COVID-19 pandemic. However, results revealed that average levels of anxiety and depressive symptoms significantly decreased from Wave 1 to Wave 8, suggesting that psychological distress waned eight weeks into the pandemic. Effect sizes indicate that this decrease was slightly stronger for anxiety symptoms (η_p_^2^ = 0.045) than for depressive symptoms (η_p_^2^ = 0.019) based on Cohen’s benchmarks of small (η_p_^2^ = .0099), medium (η_p_^2^ = .0588), and large (η_p_^2^ = .1379) effects [49, 50].

Findings also provide insight into engagement with self-quarantining and social distancing behaviors at the individual level among U.S. adults. During Wave 2, individuals reported an average of 4.70 days of self-quarantining over the prior week. At Wave 8, participants reported an average of 4.14 days of self-quarantining over the prior week. Levels of engagement in social distancing at Wave 1 and Wave 8 were comparable. ANOVAs indicated that self-quarantining generally decreased over time whereas there was a stark reduction in social distancing at Wave 7. Of note, the effect size for self-quarantining was small (η_p_^2^ = 0.014), whereas the effect size for social distancing was minimal (η_p_^2^ = 0.003). Potentially, with the loosening of COVID-19 preventive policies, people may have viewed self-quarantining as less of a necessary action, whereas they may have become more accustomed to engaging in habitual social distancing. Indeed, reductions in self-quarantining, anxiety symptoms, and depressive symptoms from Waves 1 and 2 to Wave 8 are striking given how early this was in the pandemic.

Bivariate correlations provide cross-sectional evidence that greater engagement in both self-quarantining and social distancing is associated with greater anxiety and depressive symptoms. As discussed previously, prior research provides mixed findings regarding the associations of self-quarantining and social distancing with symptoms of anxiety and depression, with some researchers finding significant positive associations [15, 24] and others finding non-significant associations [17, 18]. Inconsistencies could be due to several factors such as variability in sample sizes, sample demographics, and time of data collection. Thus, the significant correlations should be interpreted in the context of the present sample (a large national sample of 1,011 U.S. adults) and time period (March 20 to May 17, 2020).

The present findings overcome methodological limitations of prior cross-sectional and two-point longitudinal research by isolating between- and within-person effects, allowing conclusions about associations of self-quarantining and social distancing with symptoms of anxiety and depression at the group and individual level. Broadly, the present findings suggest that those who self-quarantine or social distance more also report greater levels of anxiety and depression than those who self-quarantine or social distance less (βs = .14 –.30). Effect sizes indicate that these between-person associations are large, based on guidelines for small (β = .03), medium (β = .07), and large (β = .12) effects for standardized coefficients in cross-lagged models [51]. However, associations at the within-person level indicate that more social distancing, but not self-quarantining, for a given week relative to an individual’s own average across the study was associated with greater symptoms of anxiety and depression that week relative to their own average across the study. Though the between-person associations were stronger than the within-person associations, the within-person results revealed notable small-medium effect sizes (βs = .05). Perhaps at the individual level, something more complex may be occurring when one self-quarantines. Although social isolation is generally associated with negative consequences, it is also keeping one safe from being sick or infecting others. Thus, the detrimental effects of self-quarantining may be buffered by the positive consequences (e.g., limiting exposure) of quarantine. It is also possible that individuals may not have felt fully isolated when self-quarantining given technologies that allow for social connection [52].

Although findings highlight several between-person and weekly within-person associations between social distancing and mental health symptoms, as well as between-person associations between self-quarantining and mental health, there was little evidence of weekly cross-lagged associations. These findings suggest that mental health consequences of self-quarantining and social distancing may be more transient, impacting individuals in the moment but not necessarily carrying over to have effects on subsequent weeks of functioning. The lack of evidence for prospective, predictive associations may indicate that potential third variables are at play. Other pandemic stressors may be more salient when thinking about mental health, such as job insecurity and economic concerns [18] or workplace characteristics (e.g., frontline worker position) [53]. Alternatively, factors related to the innate human capacity for adaptability and resilience may be at play [54]. Indeed, cross-sectional research has identified resiliency factors, such as hope [55] and coping flexibility [56], to have protective effects on psychological functioning in the context of the COVID-19 pandemic. Utilizing longitudinal analyses to identify pandemic and individual-related variables associated with the link between self-quarantining, social distancing, and mental health will inform interventions targeting anxiety and depression throughout the COVID-19 or future pandemic.

### Limitations

These findings should be considered in the context of some limitations. Although the design of this study allowed for dynamic longitudinal analyses of within-person, between-person, and cross lagged effects, causal claims cannot be made and there may be underlying effects of third variables (e.g., loneliness, online social connection) [57, 58]. While experimental designs can help elucidate causality and third variables, it would be unethical to implement an experimental design that involves enforced isolation and social disconnection. Further, despite recruitment of a large national sample, the sample was limited to those involved with the panel provider and was not nationally representative. Lastly, the present study’s weekly assessment structure limits the ability to test temporal effects on a smaller scale (e.g., daily basis or within-day fluctuations). Ecological momentary assessments could provide insight into smaller scale temporal fluctuations between self-quarantining, social distancing, and mental health.

## Conclusion

This study is a novel longitudinal analysis of associations between self-quarantining, social distancing, and symptoms of anxiety and depression among a U.S. sample during an 8-week period around the introduction and loosening of preventive COVID-19 policies. Previous correlational research suggests associations between self-quarantining, social distancing, and poor mental health at the group level (e.g., people who self-quarantine more report greater depression). Findings confirm between-person (group) associations of self-quarantining and social distancing with symptoms of anxiety and depression. Results also provide evidence for within-person effects indicating that when an individual engaged in greater social distancing compared to their own average, they also reported greater anxiety and depression relative to their own average. Future research using longitudinal, within-subject methods is needed to understand other factors that may affect associations between self-quarantining, social distancing, anxiety, and depression. Such information would help guide policies and interventions related to behavioral and mental health throughout the COVID-19 pandemic and future pandemics.

## Supporting information

S1 Appendix List of all study measures. (DOCX)

S1 Table Severity of anxiety and depression using imputed data. (DOCX)

S2 Table Severity of anxiety and depression using non-imputed data. (DOCX)

S3 Table Means (standard deviations) for repeated-measures ANOVA using non-imputed data. (DOCX)

S4 Table Bivariate correlations between study measures using imputed data. (XLSX)

S5 Table Bivariate correlations between study measures using non-imputed data. (XLSX)

S6 Table Fixed-effect autoregressive cross-lagged model fit statistics using non-imputed data. (DOCX)

S7 Table Standardized and unstandardized estimates for models using non-imputed data. (DOCX)
