# Supplementary material for: Self-quarantining, social distancing, and mental health during the COVID-19 pandemic: A multi wave, longitudinal investigation
Source: PLoS One. 2024 Feb 26;19(2):e0298461. doi: 10.1371/journal.pone.0298461 (PMC10896532; doi:10.1371/journal.pone.0298461)
Supplement: S1 Appendix — (DOCX) [file pone.0298461.s001.docx]

**S1 Appendix. List of all study measures.**

- Mood – 2 items
- Right Wing Authoritarianism – short form (Bizumic & Duckitt, 2018)
- Dangerous World Beliefs (Altemeyer, 1988)
- Perceived Vulnerability to Disease (Duncan et al., 2009)
- Three Domains of Disgust Scale (TDDS) – Pathogen Disgust Only (Tybur et al., 2009)
- Last 24 Hours – Disgust Propensity, Contamination Fear, Illness Recency, Face Touch
- Sleep Quality
- Last Week Health Behaviors (partially adapted from Olatunji et al. 2011)
- Past Day Health Behaviors (adapted from Olatunji et al., 2011)
- Feeling Thermometer – Social Groups
- Feeling Thermometer – Countries
- Political Ideology Scale (Shook & Clay, 2011)
- Coronavirus Questions (items developed by Shook Lab)
- Consumer Behavior
- Big Five Inventory – 10 item scale (Big 5 – BFI10; Rammstedt & John, 2007)
- Anti-Asian Prejudice
- PHQ-8 (Kroenke et al., 2001)
- GAD-7 (Spitzer et al., 2006)
- Perceived Health
- Illness Recency
- Vaccination Status
- Family Health History Questions
- Demographics
