# Supplementary material for: Self-quarantining, social distancing, and mental health during the COVID-19 pandemic: A multi wave, longitudinal investigation
Source: PLoS One. 2024 Feb 26;19(2):e0298461. doi: 10.1371/journal.pone.0298461 (PMC10896532; doi:10.1371/journal.pone.0298461)
Supplement: S1 Table — (DOCX) [file pone.0298461.s002.docx]

**S1 Table. Severity of anxiety and depression using imputed data**

| **Anxiety (GAD-7)^a^** | **Minimal - none** | **Mild** | **Moderate** | **Severe** |  | **Score ≥ 10** |
| --- | --- | --- | --- | --- | --- | --- |
| Wave 1 | 48.20% | 24.70% | 15.60% | 11.60% |  | 27.20% |
| Wave 2 | 53.20% | 23.50% | 13.20% | 10.40% |  | 23.60% |
| Wave 3 | 53.30% | 23.60% | 13.00% | 10.30% |  | 23.30% |
| Wave 4 | 53.90% | 22.50% | 14.20% | 9.90% |  | 24.10% |
| Wave 5 | 56.00% | 23.60% | 12.70% | 7.90% |  | 20.60% |
| Wave 6 | 60.70% | 20.60% | 12.40% | 6.70% |  | 19.10% |
| Wave 7 | 62.00% | 21.40% | 10.20% | 6.70% |  | 16.90% |
| Wave 8 | 60.60% | 21.10% | 10.00% | 8.50% |  | 18.50% |
|  |  |  |  |  |  |  |
| **Depression (PHQ-8)^b^** | **Minimal** | **Mild** | **Moderate** | **Moderately severe** | **Severe** | **Score ≥ 10** |
| Wave 1 | 54.20% | 20.50% | 15.00% | 6.90% | 3.70% | 25.60% |
| Wave 2 | 55.40% | 19.90% | 14.90% | 6.30% | 3.80% | 25.00% |
| Wave 3 | 56.00% | 18.60% | 12.40% | 8.60% | 4.70% | 25.70% |
| Wave 4 | 56.20% | 22.50% | 11.50% | 5.70% | 4.20% | 21.40% |
| Wave 5 | 60.20% | 18.00% | 11.70% | 6.40% | 3.90% | 22.00% |
| Wave 6 | 61.60% | 16.20% | 12.60% | 7.40% | 2.50% | 22.50% |
| Wave 7 | 62.10% | 18.30% | 10.80% | 5.90% | 3.10% | 19.80% |
| Wave 8 | 60.90% | 19.70% | 11.50% | 4.20% | 4.00% | 19.70% |

*Note*. *N* = 1,011.

^a^ GAD-7 severity ratings: minimal to no anxiety symptoms (0-4), mild anxiety symptoms (5-9), moderate anxiety symptoms (10-14), and severe anxiety symptoms (15+). Scores of ≥10 suggest presence of GAD.

^b^ PHQ-8 severity ratings: minimal depressive symptoms (0-4), mild depressive symptoms (5-9), moderate depressive symptoms (10-14), moderately severe depressive symptoms (15-19), and severe depressive symptoms (20-24). Scores of ≥10 suggest presence of major depression.
