# Supplementary material for: Self-quarantining, social distancing, and mental health during the COVID-19 pandemic: A multi wave, longitudinal investigation
Source: PLoS One. 2024 Feb 26;19(2):e0298461. doi: 10.1371/journal.pone.0298461 (PMC10896532; doi:10.1371/journal.pone.0298461)
Supplement: S2 Table — (DOCX) [file pone.0298461.s003.docx]

**S2 Table. Severity of anxiety and depression using non-imputed data.**

| **Anxiety (GAD-7)^a^** | **Minimal - none** | **Mild** | **Moderate** | **Severe** |  | **Score ≥ 10** |
| --- | --- | --- | --- | --- | --- | --- |
| Wave 1 | 48.10% | 24.70% | 15.60% | 11.60% |  | 27.20% |
| Wave 2 | 45.00% | 19.10% | 10.30% | 7.70% |  | 18.00% |
| Wave 3 | 43.40% | 16.90% | 9.70% | 6.60% |  | 16.30% |
| Wave 4 | 41.60% | 16.80% | 9.30% | 6.00% |  | 15.30% |
| Wave 5 | 42.70% | 16.20% | 8.10% | 4.60% |  | 12.70% |
| Wave 6 | 43.40% | 13.90% | 7.40% | 3.80% |  | 11.20% |
| Wave 7 | 38.70% | 12.00% | 5.80% | 3.20% |  | 9.00% |
| Wave 8 | 42.20% | 13.20% | 5.40% | 4.10% |  | 9.50% |
|  |  |  |  |  |  |  |
| **Depression (PHQ-8)^b^** | **Minimal** | **Mild** | **Moderate** | **Moderately severe** | **Severe** | **Score ≥ 10** |
| Wave 1 | 54.10% | 20.50% | 15.00% | 6.90% | 3.70% | 25.60% |
| Wave 2 | 46.70% | 15.50% | 11.40% | 5.00% | 3.20% | 19.60% |
| Wave 3 | 44.90% | 14.50% | 8.30% | 6.10% | 2.70% | 17.10% |
| Wave 4 | 43.30% | 16.30% | 8.10% | 3.90% | 2.30% | 14.30% |
| Wave 5 | 45.00% | 13.50% | 7.00% | 4.00% | 2.10% | 13.10% |
| Wave 6 | 44.00% | 11.70% | 7.40% | 3.90% | 1.30% | 12.60% |
| Wave 7 | 38.70% | 11.20% | 5.30% | 3.00% | 1.70% | 10.00% |
| Wave 8 | 41.60% | 13.40% | 5.90% | 2.20% | 1.90% | 10.00% |

*Note*. *N* = 602-1,010.

^a^ GAD-7 severity ratings: minimal to no anxiety symptoms (0-4), mild anxiety symptoms (5-9), moderate anxiety symptoms (10-14), and severe anxiety symptoms (15+). Scores of ≥10 suggest presence of GAD.

^b^ PHQ-8 severity ratings: minimal depressive symptoms (0-4), mild depressive symptoms (5-9), moderate depressive symptoms (10-14), moderately severe depressive symptoms (15-19), and severe depressive symptoms (20-24). Scores of ≥10 suggest presence of major depression.
