# Supplementary material for: Self-quarantining, social distancing, and mental health during the COVID-19 pandemic: A multi wave, longitudinal investigation
Source: PLoS One. 2024 Feb 26;19(2):e0298461. doi: 10.1371/journal.pone.0298461 (PMC10896532; doi:10.1371/journal.pone.0298461)
Supplement: S3 Table — (DOCX) [file pone.0298461.s004.docx]

**S3 Table. Means (standard deviations) for repeated-measures ANOVA using non-imputed data.**

|  | **Wave 1** | **Wave 2** | **Wave 3** | **Wave 4** | **Wave 5** | **Wave 6** | **Wave 7** | **Wave 8** |
| --- | --- | --- | --- | --- | --- | --- | --- | --- |
| Self-quarantining^a^ | — | 4.68  (2.88)^7,8^ | 4.54  (2.95)^8^ | 4.70  (2.90)^6,7,8^ | 4.63  (2.88)^7,8^ | 4.41  (3.01)^4^ | 4.25  (3.06)^2,4,5^ | 4.20  (2.99)^2,3,4,5^ |
| Social Distancing^b^ | -0.07  (0.67) | -0.03  (0.66) | -0.02  (0.69) | -0.03  (0.68) | -0.004  (0.70) | -0.03  (0.70) | -0.03 (0.70) | -0.001  (0.70) |
| Anxiety^c^ | 5.09 (5.43)^4,5,6,7,8^ | 4.72 (5.43)^5,6,7,8^ | 4.73 (5.42)^5,6,7,8^ | 4.54 (5.37)^1,5,6,7,8^ | 4.13 (5.30)^1,2,3,4,6,7,8^ | 3.83 (5.09)^1,2,3,4,5^ | 3.59 (4.95)^1,2,3,4,5^ | 3.67 (5.09)^1,2,3,4,5^ |
| Depression^d^ | 4.81 (5.54)^6,7,8^ | 4.73 (5.48)^6,8^ | 4.85 (5.73)^5,6,7,8^ | 4.69 (5.61)^6,8^ | 4.45 (5.60)^3,8^ | 4.20 (5.42)^1,2,3,4^ | 4.33 (5.78)^1,3^ | 4.09 (5.38)^1,2,3,4,5^ |

*Note*. Superscripts indicate significant mean differences between weeks (e.g., ^7,8^ above Wave 2 for self-quarantining indicates that the mean for Wave 2 self-quarantining is significantly higher than the means for Waves 7 and 8 self-quarantining).

^a^ Self-quarantining was not assessed at Wave 1. There was a significant main effect of time for self-quarantining, *F*(5.28, 2012.84) = 4.50, *p* < 0.001, η_p_^2^ = 0.012.

^b^ The social distancing composite reflects six items which were standardized and averaged to have a mean of 0 and standard deviation of 1. The main effect of time for social distancing was not significant, *F*(6.36, 2703.26) = 1.13, *p* = 0.34, η_p_^2^ = 0.003.

^c^ There was a significant main effect of time for anxiety, *F*(5.72, 2354.16) = 17.88, *p* < 0.001, η_p_^2^ = 0.041.

^d^ There was a significant main effect of time for depression, *F*(6.13, 2526.85) = 4.51, *p* < 0.001, η_p_^2^ = 0.011.
