# Supplementary material for: Self-quarantining, social distancing, and mental health during the COVID-19 pandemic: A multi wave, longitudinal investigation
Source: PLoS One. 2024 Feb 26;19(2):e0298461. doi: 10.1371/journal.pone.0298461 (PMC10896532; doi:10.1371/journal.pone.0298461)
Supplement: S6 Table — (DOCX) [file pone.0298461.s007.docx]

**S6 Table. Fixed-effect autoregressive cross-lagged model fit statistics using non-imputed data.**

| Model | χ^2^ (df) | CFI | TLI | RMSEA [90% CI] | SRMR |
| --- | --- | --- | --- | --- | --- |
| Self-quarantining |  |  |  |  |  |
| Anxiety symptoms | 200.55 (92) | 0.97 | 0.98 | 0.06 [0.05, 0.07] | 0.05 |
| Depressive symptoms | 201.20 (92) | 0.97 | 0.98 | 0.06 [0.05, 0.07] | 0.05 |
| Social distancing |  |  |  |  |  |
| Anxiety symptoms | 342.78 (123) | 0.96 | 0.96 | 0.07 [0.06, 0.08] | 0.06 |
| Depressive symptoms | 314.87 (123) | 0.97 | 0.97 | 0.06 [0.06, 0.07] | 0.06 |

*Note*. *N* = 345-393. All *χ*^2^ values are significant at *p* < .001.
