# Supplementary material for: Self-quarantining, social distancing, and mental health during the COVID-19 pandemic: A multi wave, longitudinal investigation
Source: PLoS One. 2024 Feb 26;19(2):e0298461. doi: 10.1371/journal.pone.0298461 (PMC10896532; doi:10.1371/journal.pone.0298461)
Supplement: S7 Table — (DOCX) [file pone.0298461.s008.docx]

**S7 Table. Standardized and unstandardized estimates for models using non-imputed data.**

|  | Mental Health | | | | | | | | | | |
| --- | --- | --- | --- | --- | --- | --- | --- | --- | --- | --- | --- |
|  | Anxiety Symptoms | | | |  | Depressive Symptoms | | | | | |
|  | β | *B* | 90% CI | *SE* |  | β | *B* | | 90% CI | | *SE* |
| Self-quarantining (SQ) |  |  |  |  |  |  |  |  |  |  |  |
| SQ ↔︎ MH Intercept Covariance (Between-person) | 0.120 | 1.199 | [0.009, 2.388] | 1.975 |  | 0.096 | 1.033 | [-0.236, 2.302] | | 0.648 | |
| SQ ↔︎ MH Weekly Covariance (Within-person) | 0.032 | 0.142 | [-0.076, 0.360] | 0.111 |  | -0.012 | -0.055 | [-0.283, 0.172] | | 0.116 | |
| SQ Autoregressive Path | 0.21 | 0.21** | [0.158, 0.265] | 0.03 |  | 0.20 | 0.20** | [0.147, 0.255] | | 0.03 | |
| MH Autoregressive Path | 0.28 | 0.28** | [0.227, 0.334] | 0.03 |  | 0.20 | 0.20** | [0.140, 0.249] | | 0.03 | |
| SQ → MH Weekly Cross-Lagged | -0.024 | -0.031 | [-0.093, 0.031] | 0.032 |  | 0.006 | 0.008 | [-0.057, 0.074] | | 0.034 | |
| MH → SQ Weekly Cross-Lagged | -0.002 | -0.001 | [-0.039, 0.036] | 0.019 |  | 0.026 | 0.019 | [-0.017, 0.055] | | 0.018 | |
|  |  |  |  |  |  |  |  |  | |  | |
| Social Distancing (SD) |  |  |  |  |  |  |  |  | |  | |
| SD ↔︎ MH Intercept Covariance (Between-person) | 0.263 | 0.660** | [0.386, 0.935] | 0.140 |  | 0.256 | 0.695** | [0.400, 0.989] | | 0.150 | |
| SD ↔︎ MH Weekly Covariance (Within-person) | 0.055 | 0.054* | [0.013, 0.094] | 0.021 |  | 0.030 | 0.031 | [-0.012, 0.074] | | 0.022 | |
| SD Autoregressive Path | 0.10 | 0.10** | [0.057, 0.143] | 0.02 |  | 0.09 | 0.09** | [0.050, 0.136] | | 0.02 | |
| MH Autoregressive Path | 0.22 | 0.22** | [0.176, 0.263] | 0.02 |  | 0.19 | 0.19** | [0.142, 0.231] | | 0.02 | |
| SD → MH Weekly Cross-Lagged | -0.018 | -0.099 | [-0.377, 0.179] | 0.142 |  | -0.029 | -0.216 | [-0.506, 0.074] | | 0.144 | |
| MH → SD Weekly Cross-Lagged | 0.013 | 0.001 | [-0.004, 0.007] | 0.003 |  | 0.004 | 0.000 | [-0.005, 0.006] | | 0.003 | |

*Note*. *N* = 345-393. MH = Mental Health.

* *p* < .01. ** *p* < .001.
